# Metformin use history and genome-wide DNA methylation profile: potential molecular mechanism for aging and longevity

**DOI:** 10.18632/aging.204498

**Published:** 2023-02-02

**Authors:** Pedro S. Marra, Takehiko Yamanashi, Kaitlyn J. Crutchley, Nadia E. Wahba, Zoe-Ella M. Anderson, Manisha Modukuri, Gloria Chang, Tammy Tran, Masaaki Iwata, Hyunkeun Ryan Cho, Gen Shinozaki

**Affiliations:** 1 Department of Psychiatry and Behavioral Sciences, Stanford University School of Medicine, Palo Alto, CA 94304, USA; 2 Department of Psychiatry, University of Iowa Carver College of Medicine, Iowa City, IA 52242, USA; 3 Department of Neuropsychiatry, Tottori University Faculty of Medicine, Yonago-shi, Tottori 680-8550, Japan; 4 University of Nebraska Medical Center College of Medicine, Omaha, NE 68131, USA; 5 Department of Psychiatry, Oregon Health and Science University School of Medicine, Portland, OR 97239, USA; 6 Department of Biostatistics, University of Iowa College of Public Health, Iowa City, IA 52242, USA

**Keywords:** metformin, longevity, diabetes, epigenetics, aging, inflammation, methylation

## Abstract

Background: Metformin, a commonly prescribed anti-diabetic medication, has repeatedly been shown to hinder aging in pre-clinical models and to be associated with lower mortality for humans. It is, however, not well understood how metformin can potentially prolong lifespan from a biological standpoint. We hypothesized that metformin’s potential mechanism of action for longevity is through its epigenetic modifications.

Methods: To test our hypothesis, we conducted a post-hoc analysis of available genome-wide DNA methylation (DNAm) data obtained from whole blood collected from inpatients with and without a history of metformin use. We assessed the methylation profile of 171 patients (first run) and only among 63 diabetic patients (second run) and compared the DNAm rates between metformin users and nonusers.

Results: Enrichment analysis from the Kyoto Encyclopedia of Genes and Genome (KEGG) showed pathways relevant to metformin’s mechanism of action, such as longevity, AMPK, and inflammatory pathways. We also identified several pathways related to delirium whose risk factor is aging. Moreover, top hits from the Gene Ontology (GO) included HIF-1α pathways. However, no individual CpG site showed genome-wide statistical significance (*p* < 5E-08).

Conclusion: This study may elucidate metformin’s potential role in longevity through epigenetic modifications and other possible mechanisms of action.

## INTRODUCTION

We live in an aging society. According to the U.S. Census Bureau’s 2017 National Population Projections, 1 in every 5 residents will be in retirement age by 2030 [1]. Subsequently, a more significant percentage of the population will endure the challenges of age-related diseases than ever before. Treatments targeting these diseases, such as dementia or cancer, at most “delay” the disease process but have a limited ability to “cure.” Therefore, there are growing interests in treating aging itself as a disease [2].

Considerable evidence from basic and pre-clinical models shows that several interventions, such as exercise, intermittent fasting, and even ingestion of certain compounds can prolong lifespan. These promising compounds include rapamycin [3, 4], resveratrol [5–7], NAD [8], and metformin [9–11]. Our group also confirmed that inpatients using metformin had improved three-year survival rates compared to non-metformin users [12]. In addition, our data also showed that prevalence of delirium was lower among those who were on metformin compared to those without [12].

The mechanism (or mechanisms) of action that rationalizes how these interventions prolong lifespan, or potentially delay aging, has been investigated heavily. Nevertheless, no exact process is well understood, especially for metformin. It is believed that epigenetics is one of the most important molecular mechanisms of aging in animals and plants; thus, it is plausible that the “life-prolonging” effects of many interventions are through modification of epigenetic processes. For example, several reports show epigenetic changes from exercise [13], fasting [14], rapamycin [3], resveratrol [5], and NAD [8]. However, there are only a few studies investigating the direct influence of metformin on epigenetic changes [15–17], suggesting that information about the influence of metformin on the epigenetic profile in humans is currently limited.

To fill such gap of knowledge, we investigated the potential influence of metformin on the epigenetic profile by testing genome-wide DNA methylation (DNAm) in whole blood samples obtained from inpatients with and without a history of metformin use.

## RESULTS

### Demographics

173 subjects were enrolled in this study, but only 171 were included in downstream data analysis. The average patient age was 74.4 (SD = 9.8). 58 (33.9%) subjects were females while almost all the subjects were white per self-report (*n* = 167; 97.7%). 108 patients were non-diabetic (non-DM) while 63 were diabetic (DM). Among the DM group, 37 had diabetes with a history of metformin prescription DM(+)Met and 26 had diabetes without a history of metformin prescription DM(−)Met. Additionally, 43 (68.3%) diabetic subjects had a history of insulin use. Charlson Comorbidity Index (CCI) and body mass index (BMI) information are also included in Table 1. No variable revealed statistically significant differences between the DM(−)Met and DM(+)Met. However CCI, BMI, and insulin use were significantly higher among the DM group compared to the non-DM group, as expected.

**Table 1:** Patient characteristics.

**Classification**	**All Subjects**	**Diabetes**		***p***	**Statistical test**	**DM subjects**		***p***	**Statistical test**
		**non-DM**	**DM**			**DM(−)Met**	**DM(+)Met**		
*N*	171	108	63			26	37		
Age - yr	74.4	74.6	74.1	0.77	*t* = 1.98	73.8	74.3	0.833	*t* = 2.01
SD	9.8	9.7	10.0			10.6	9.7		
Female sex (*n*)	58	36	22	0.81	χ^2^ = 0.10	11	11	0.303	χ^2^ = 1.06
%	33.9	33.6	34.9			42.3	29.7		
Race, White (*n*)	167	105	62	0.63	χ^2^ = 0.23	25	37	0.229	χ^2^ = 1.45
%	97.7	97.2	98.4			96.2	100		
CCI	3.8	3.1	4.9	7.5E-06^*^	*t* = 1.98	4.8	5.0	0.756	*t* = 2.00
SD	2.7	2.7	2.4			2.4	2.5		
BMI	29.7	28.3	32.2	0.002^*^	*t* = 1.98	30.0	33.8	0.64	*t* = 2.00
SD	7.6	6.3	8.8			5.0	10.5		
Insulin use history	43	0	43	3.3E-23^*^	χ^2^ = 98.48	15	28	0.131	χ^2^ = 2.28
%	25.1	0	68.3			57.7	75.7		

Age, sex, and race were not significantly different between the non-diabetes (non-DM) and the diabetes (DM) groups, while CCI, BMI, and insulin use were. None of the patient characteristics between metformin nonusers DM(−)Met and metformin users DM(+)Met among the diabetic group were statistically significant. Abbreviations: SD: Standard deviation; CCI: Charlson comorbidity index; BMI: Body mass index. ^*^p < 0.05.

### Met vs. non-Met (including all patients regardless of diabetes status): top hits, KEGG, GO

Table 2 shows the most significant genes that differed in methylation rates between patients with and without metformin use history regardless of diabetes status (171 subjects). None of the sites met the criteria for genome-wide statistical significance (*p* < 5E-8).

**Table 2:** Top 20 CpG sites that differed between metformin users and nonusers among all patients.

**Gene name**	**CpG site**	**Chromosome**	**non-Met (%)**	**Met (%)**	**% mean difference (Δβ)**	***p*-value**
PSME3	cg22769787	chr17	15.6%	14.3%	1.3%	3.37E-07
EPHA8	cg27136384	chr1	83.2%	−2.7%	−2.7%	4.84E-07
	cg22163972	chr17	92.1%	4.2%	4.2%	4.89E-07
	cg23047680	chr3	0.8%	−0.2%	−0.2%	9.08E-07
NEDD4	cg11341892	chr15	4.7%	0.6%	0.6%	2.82E-06
PRKCG	cg11293016	chr19	52.9%	4.0%	4.0%	4.68E-06
SRSF11	cg12923877	chr1	97.5%	−0.3%	−0.3%	4.94E-06
RRP15	cg24353272	chr1	95.3%	−0.8%	−0.8%	5.16E-06
KIAA1688	cg07969649	chr8	91.1%	−1.6%	−1.6%	5.22E-06
TRIM27	cg02525926	chr6	97.4%	0.8%	0.8%	6.98E-06
	cg23067796	chr12	93.7%	1.7%	1.7%	7.29E-06
RYR2	cg04573831	chr1	96.6%	−0.6%	−0.6%	8.11E-06
	cg15180899	chr18	93.9%	1.7%	1.7%	8.67E-06
	cg12222244	chr3	94.1%	2.1%	2.1%	1.27E-05
C1orf125	cg20746459	chr1	90.6%	3.5%	3.5%	1.52E-05
SERPINH1	cg19586851	chr11	97.2%	−0.5%	−0.5%	1.55E-05
PPL	cg12991522	chr16	1.8%	−0.5%	−0.5%	1.55E-05
ACO1	cg13567378	chr9	89.0%	−1.3%	−1.3%	1.71E-05
	cg24525630	chr17	1.6%	−0.3%	−0.3%	1.72E-05
TCF7L1	cg20116596	chr2	95.7%	−0.5%	−0.5%	1.76E-05

Next, we conducted enrichment analysis using the top 330 CpG sites based on the absolute difference in methylation level (beta value) between metformin users (Met) and nonusers (non-Met) greater than 4% and the *p*-value less than 0.01. Enrichment analysis from the KEGG top signals showed relevant pathways to metformin’s possible roles, such as “longevity regulating pathway”, “longevity regulating pathway – multiple species”, and “AMPK signaling pathway” (Table 3). In addition, other pathways, such as “mTOR signaling pathway”, “insulin secretion”, “glutamatergic synapse”, and “circadian entrainment” were discovered (Table 3). There were also relevant pathways revealed in the GO analysis, such as “regulation of hypoxia-inducible factor-1alpha signaling pathway”, “positive regulation of hypoxia-inducible factor-1alpha signaling pathway”, and “canonical Wnt signal pathway” (Table 4), although none of the pathways in either KEGG or GO reached the False Discovery Rate (FDR) significance level (FDR <0.05) (Tables 3 and 4).

**Table 3:** Top 30 KEGG pathways based on different methylation rates between metformin users and nonusers.

**Pathway**	***N***	**DE**	***p*-value**	**FDR**
Relaxin signaling pathway	129	6	0.007	1
**Longevity regulating pathway**	89	5	0.008	1
**Glutamatergic synapse**	114	6	0.008	1
Cushing syndrome	155	6	0.018	1
Parathyroid hormone synthesis, secretion and action	106	5	0.019	1
**AMPK signaling pathway**	119	5	0.021	1
Signaling pathways regulating pluripotency of stem cells	142	5	0.028	1
Gap junction	88	4	0.033	1
Insulin secretion	86	4	0.034	1
Melanogenesis	101	4	0.043	1
**Longevity regulating pathway - multiple species**	62	3	0.051	1
Aldosterone synthesis and secretion	98	4	0.055	1
Chemical carcinogenesis - DNA adducts	69	2	0.056	1
**Circadian entrainment**	97	4	0.058	1
Steroid hormone biosynthesis	61	2	0.062	1
Thermogenesis	219	5	0.063	1
Bile secretion	89	3	0.063	1
Metabolism of xenobiotics by cytochrome P450	76	2	0.068	1
Cortisol synthesis and secretion	65	3	0.069	1
Thyroid hormone synthesis	75	3	0.071	1
Wnt signaling pathway	166	5	0.071	1
Vasopressin-regulated water reabsorption	44	2	0.081	1
**Cholinergic synapse**	113	4	0.090	1
Retrograde endocannabinoid signaling	141	4	0.089	1
Estrogen signaling pathway	137	4	0.091	1
Mineral absorption	60	2	0.111	1
Gastric cancer	149	4	0.121	1
**mTOR signaling pathway**	155	4	0.123	1
Protein digestion and absorption	102	3	0.123	1
Ovarian steroidogenesis	51	2	0.123	1
Thyroid hormone synthesis	75	3	0.071	1

Relevant pathways from KEGG [58] are highlighted. Abbreviations: *N*: number of genes included in each pathway; DE: number of Differentially Expressed genes, which are the number of genes from the top CpG sites; FDR: False Discovery Rate.

**Table 4:** Top 30 GO pathways based on different methylation rates between metformin users and nonusers.

**Pathway**	**Ont**	***N***	**DE**	***p*-value**	**FDR**
Homophilic cell adhesion via plasma membrane adhesion molecules	BP	168	8	7.20E-04	1
Long-term synaptic depression	BP	31	4	9.77E-04	1
Locomotory behavior	BP	198	9	0.001	1
Midbrain dopaminergic neuron differentiation	BP	17	3	0.002	1
Cell surface receptor signaling pathway involved in cell-cell signaling	BP	622	17	0.002	1
Negative regulation of synaptic transmission	BP	71	5	0.002	1
Canonical Wnt signaling pathway	BP	335	11	0.002	1
Calcium ion binding	MF	698	17	0.003	1
Hexose mediated signaling	BP	6	2	0.003	1
Sugar mediated signaling pathway	BP	6	2	0.003	1
Glucose mediated signaling pathway	BP	6	2	0.003	1
Cellular response to acid chemical	BP	209	8	0.003	1
Cell-cell signaling	BP	1847	345	0.003	1
Regulation of ion transmembrane transporter activity	BP	256	9	0.004	1
Mesoderm development	BP	133	6	0.004	1
Neuronal cell body membrane	CC	27	3	0.004	1
Cell body membrane	CC	28	3	0.004	1
Regulation of transmembrane transporter activity	BP	264	9	0.005	1
**Regulation of hypoxia-inducible factor-1alpha signaling pathway**	BP	1	1	0.005	1
**Positive regulation of hypoxia-inducible factor-1alpha signaling pathway**	BP	1	1	0.005	1
Cellular response to vitamin K	BP	1	1	0.005	1
Cellular response to glucagon stimulus	BP	25	3	0.005	1
Carbohydrate mediated signaling	BP	8	2	0.005	1
Seminal vesicle morphogenesis	BP	1	1	0.005	1
Glucagon-like peptide 1 receptor activity	MF	1	1	0.005	1
Behavior	BP	593	15	0.005	1
Nicotinamide phosphoribosyltransferase activity	MF	1	1	0.006	1
Response to D-galactose	BP	1	1	0.006	1
Embryonic skeletal system development	BP	125	6	0.006	1
Regulation of transporter activity	BP	279	9	0.006	1

Relevant pathways are highlighted. Abbreviations: Ont: Ontology; BP: biological process; CC: cellular component; MF: molecular function; *N*: number of genes included in each pathway; DE: number of Differentially Expressed genes, which are the number of genes from the top CpG sites; FDR: False Discovery Rate.

### Met vs. non-Met (including only patients with type 2 diabetes mellitus): top hits, KEGG, GO

Table 5 shows the most significant genes that differed in methylation rate between metformin users and nonusers among the diabetes group (63 subjects). Similar to the previous analysis, no gene reached genome-wide statistical significance (*p* < 5E-8).

**Table 5:** Top 20 CpG sites that differed between metformin users and nonusers among the diabetes group.

**Gene name**	**CpG site**	**Chromosome**	**non-Met (%)**	**Met (%)**	**Mean difference (Δβ)**	***p*-value**
	cg19873536	chr10	78.3%	67.9%	10.4%	1.28E-06
	cg13596208	chr9	1.9%	2.7%	−0.9%	2.29E-06
HBA1	cg01704105	chr16	40.5%	33.7%	6.8%	5.42E-06
DUOX2	cg02550961	chr15	1.5%	1.9%	−0.4%	6.10E-06
NEO1	cg12516231	chr15	2.2%	3.2%	−0.9%	6.97E-06
C7orf46	cg06685724	chr7	2.1%	2.9%	−0.8%	1.28E-05
NAT15	cg00484396	chr16	9.8%	4.9%	4.8%	1.56E-05
	cg14685975	chr5	89.9%	92.1%	−2.2%	1.64E-05
CTSL	cg02104500	chr9	3.6%	4.9%	−1.4%	1.66E-05
	cg12584257	chr9	67.6%	77.2%	−9.6%	1.69E-05
NAT15	cg22508957	chr16	10.9%	6.3%	4.6%	1.84E-05
AREL1	cg11034672	chr14	11.6%	15.0%	−3.3%	1.86E-05
	cg24651265	chr10	1.1%	1.7%	−0.5%	2.12E-05
CMBL	cg17467873	chr5	1.7%	2.1%	−0.4%	2.21E-05
EBF4	cg05857996	chr20	77.6%	63.6%	13.9%	2.23E-05
	cg18482666	chr2	95.8%	94.8%	1.0%	2.39E-05
HRASLS5	cg00489394	chr11	6.6%	7.1%	−0.5%	2.40E-05
AKAP13	cg21530087	chr15	2.2%	2.6%	−0.4%	2.59E-05
	cg15864571	chr3	93.4%	95.0%	−1.6%	2.67E-05
FLJ35024	cg15981195	chr9	2.3%	3.5%	−1.1%	2.91E-05

The enrichment analysis was generated using consistent parameters in methylation level differences (beta >4%) and *p*-value (<0.01). This current analysis, however, included 1283 CpGs. KEGG showed many of the same signals discovered from the previous analysis, including “longevity regulating pathway”, “glutamatergic synapse”, “insulin secretion”, “circadian entrainment”, and “cholinergic synapse” (Table 6). GO also showed overlapping pathways compared to the first analysis, including “hypoxia-inducible factor-1alpha signaling pathway”, but also new pathways, such as “interleukin-8-mediated signaling pathway”, “negative regulation of leukocyte apoptotic process”, “neutrophil homeostasis”, and “neuron projection”, although these pathways did not reach the FDR significance level (FDR <0.05) (Table 7).

**Table 6:** Top 30 KEGG pathways that differed between metformin users and nonusers among the diabetes group.

**Pathway**	***N***	**DE**	***p*-value**	**FDR**
Aldosterone synthesis and secretion	98	14	0.001	0.219
**Circadian entrainment**	97	14	0.001	0.219
Cortisol synthesis and secretion	65	10	0.003	0.303
Thyroid hormone synthesis	75	10	0.004	0.303
Regulation of lipolysis in adipocytes	55	8	0.006	0.330
Parathyroid hormone synthesis, secretion and action	106	13	0.006	0.330
Insulin secretion	86	11	0.007	0.330
Calcium signaling pathway	238	21.5	0.009	0.388
cAMP signaling pathway	221	19	0.010	0.388
**Cholinergic synapse**	113	13	0.012	0.420
Chemical carcinogenesis - receptor activation	212	16	0.015	0.435
**Glutamatergic synapse**	114	13	0.016	0.435
Rap1 signaling pathway	210	19	0.016	0.435
Thermogenesis	219	15	0.020	0.468
Amphetamine addiction	69	8	0.021	0.468
Neuroactive ligand-receptor interaction	349	19.5	0.022	0.468
Pancreatic secretion	101	9	0.029	0.552
Long-term potentiation	67	8	0.029	0.552
Cocaine addiction	49	6	0.036	0.552
Phospholipase D signaling pathway	147	14.5	0.038	0.552
cGMP-PKG signaling pathway	166	14.5	0.039	0.555
Apelin signaling pathway	139	12	0.040	0.555
Nicotine addiction	40	5	0.040	0.555
EGFR tyrosine kinase inhibitor resistance	78	9	0.041	0.555
Inflammatory mediator regulation of TRP channels	98	10	0.042	0.555
Gap junction	88	9	0.042	0.555
Type II diabetes mellitus	46	6	0.043	0.558
**Longevity regulating pathway**	89	9	0.045	0.561
Salivary secretion	92	8	0.048	0.578
Bladder cancer	41	5	0.054	0.610

Relevant pathways from KEGG [58] are highlighted. Abbreviations: *N*: number of genes included in each pathway; DE: number of Differentially Expressed genes, which are the number of genes from the top CpG sites; FDR: False Discovery Rate.

**Table 7:** Top 30 GO pathways that differed between metformin users and nonusers among the diabetes group.

**Pathway**	**Ont**	***N***	**DE**	***p*-value**	**FDR**
Neuron projection	CC	1304	97.1	1.29E-05	0.286
Second-messenger-mediated signaling	BP	438	38.5	2.53E-05	0.286
Neutrophil homeostasis	BP	16	6	3.77E-05	0.286
Synaptic signaling	BP	725	59.5	9.26E-05	0.505
Trans-synaptic signaling	BP	708	57.5	1.67E-04	0.505
Negative regulation of leukocyte apoptotic process	BP	46	8	1.93E-04	0.505
Calcium-mediated signaling	BP	218	22.5	1.94E-04	0.505
Chemical synaptic transmission	BP	700	56.5	2.00E-04	0.505
Anterograde trans-synaptic signaling	BP	700	56.5	2.00E-04	0.505
Positive regulation of cell-matrix adhesion	BP	51	10	3.21E-04	0.682
Positive regulation of multicellular organismal process	BP	1802	106	3.39E-04	0.682
Plasma membrane bounded cell projection	CC	2093	130.1	4.05E-04	0.682
Interleukin-8 receptor activity	MF	2	2	4.44E-04	0.682
Interleukin-8-mediated signaling pathway	BP	2	2	4.44E-04	0.682
Adult behavior	BP	144	17.5	4.73E-04	0.682
Cell junction	CC	1858	123.8	5.16E-04	0.682
Synapse	CC	1168	85.5	5.27E-04	0.682
NMDA glutamate receptor activity	MF	7	4	6.05E-04	0.682
**Hypoxia-inducible factor-1alpha signaling pathway**	BP	6	3	6.39E-04	0.682
Regulation of dendrite development	BP	148	19	6.44E-04	0.682
Axon	CC	606	50.6	6.46E-04	0.682
Low voltage-gated calcium channel activity	MF	3	3	7.18E-04	0.682
Dendrite development	BP	232	26.5	7.28E-04	0.682
Vestibulocochlear nerve development	BP	10	4	7.64E-04	0.682
Ionotropic glutamate receptor signaling pathway	BP	25	7	7.69E-04	0.682
Neuron projection development	BP	976	74	8.82E-04	0.682
Cellular response to glucose stimulus	BP	132	15	9.26E-04	0.682
Locomotory behavior	BP	198	21.5	9.36E-04	0.682
Cellular response to hexose stimulus	BP	134	15	1.08E-03	0.682
Positive regulation of cellular component biogenesis	BP	533	41	1.12E-03	0.682

Relevant pathways are highlighted. Abbreviations: Ont: Ontology; BP: biological process; CC: cellular component; MF: molecular function; *N*: number of genes included in each pathway; DE: number of Differentially Expressed genes, which are the number of genes from the top CpG sites; FDR: False Discovery Rate.

### DNA methylation age acceleration

**Figure 1:**
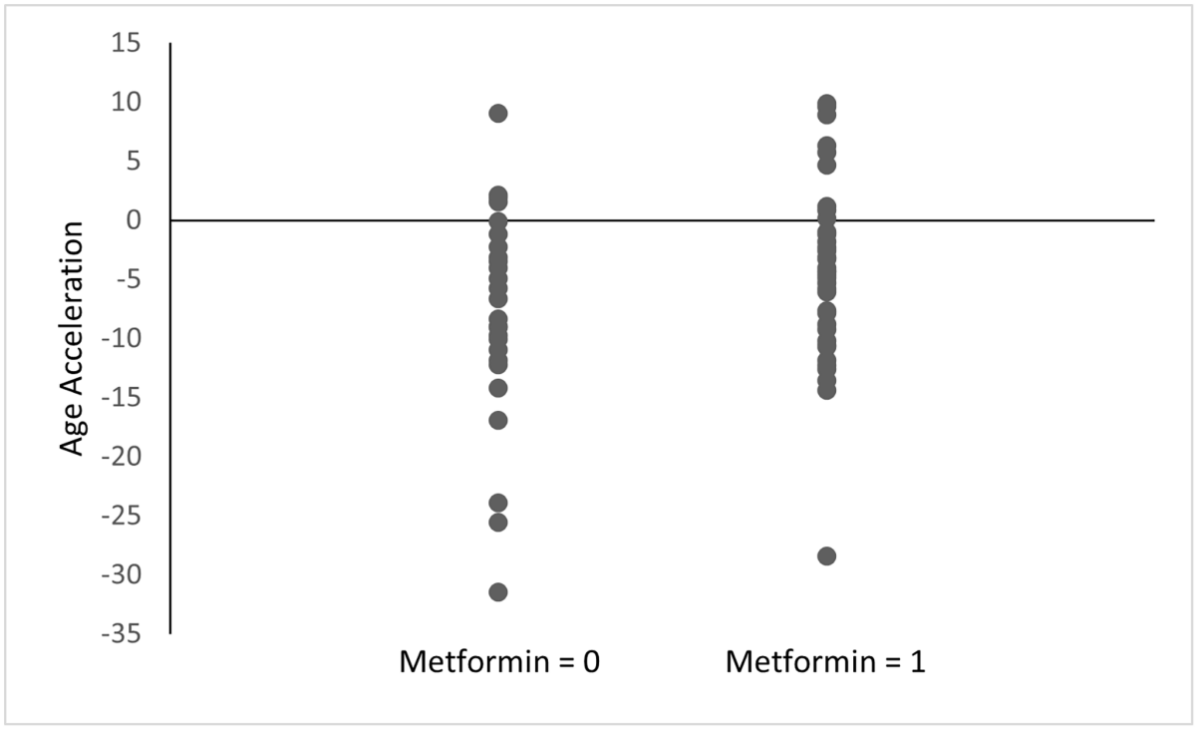
**Age acceleration between metformin users and nonusers among the diabetes group.** Age acceleration was calculated using the Horvath epigenetic clock as DNAm age - chronological age. Metformin = 0: without history of metformin use, Metformin = 1: with history of metformin use. *p* = 0.11.

**Figure 2:**
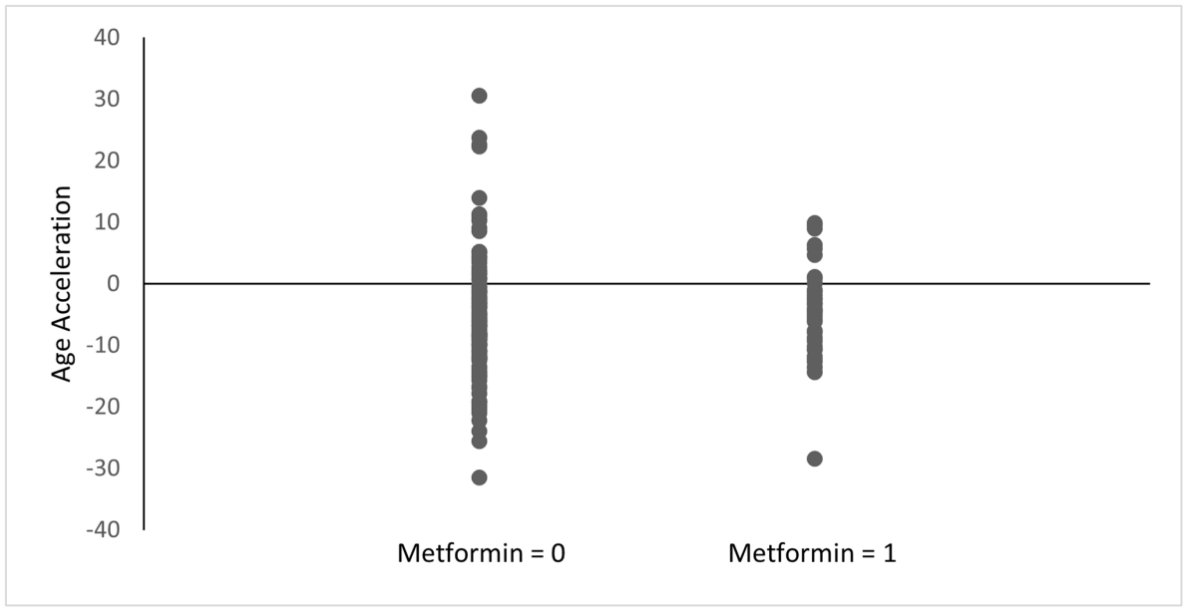
**Age acceleration between metformin users and nonusers.** Age acceleration was calculated using the Horvath epigenetic clock as DNAm age - chronological age. Metformin = 0: without history of metformin use, Metformin = 1: with history of metformin use. *p* = 0.34.

Among the diabetes group, metformin nonusers had a mean age acceleration of −8.07 compared to a mean age acceleration of −4.47 for metformin users (*p* = 0.11) (Figure 1). This difference was smaller among all the subjects included regardless of diabetes status (−5.92 for metformin nonusers vs. −4.47 for metformin users; *p* = 0.34) (Figure 2). Both analyses did not reach statistical significance.

## DISCUSSION

In this study, we compared genome-wide DNA methylation rates among metformin users and nonusers to investigate the potential epigenetic effects of metformin exposure. Enrichment analysis was employed to elucidate the possible mechanisms of action induced by metformin. Our KEGG analysis revealed evidence of differences in epigenetic profiles involved in “longevity” such as “longevity regulating pathway” and “longevity regulating pathway – multiple species” (Tables 3 and 6). Although it was not statistically significant, the appearance of these pathways among top signals in the KEGG analysis demonstrates the potential role of the epigenetic processes manifesting the effect of metformin on longevity. The same KEGG analysis also showed “AMPK signaling pathway” (Table 3). AMP-activated protein kinase (AMPK), an energy sensor that regulates metabolism, is commonly referred to as one of the targets of metformin’s hypothetical mechanisms of action [18, 19], although there is also evidence that metformin’s effects are in part AMPK-independent [20]. Furthermore, AMPK activation is related to subsequent activation of hypoxia-inducible factors [21] which also appeared in our GO analyses as “regulation of hypoxia-inducible factor-1alpha signaling pathway” and “positive regulation of hypoxia-inducible factor-1alpha signaling pathway” (Table 4), as well as “hypoxia-inducible factor-1alpha signaling pathway” (Table 7). Hypoxia-inducible factor-1alpha (HIF-1α) is a transcription factor expressed in nucleated cells and mediated by oxygen levels. HIF-1α has been implicated in age-related diseases, endothelial senescence progression, AMPK, and many other pathways [22]. Beyond metformin’s potential epigenetic medication related to longevity, several pathways related to delirium, such as “circadian entrainment”, “cholinergic synapse”, and “glutamatergic synapse”, were identified (Tables 3 and 6). These pathways are intriguing from metformin’s possible “anti-aging” standpoint as age is a major risk factor of delirium.

The beneficial effects of metformin on lifespan have been widely studied. Previous studies reported that metformin increased median lifespan of *C. elegans* co-cultured with *E.coli* by more than 35% [9, 23], and prolonged the lifespan of mice [10]. Patients with age-related diseases such as cardiovascular diseases and cancer who take metformin also had lower rates of mortality [24, 25]. Our recent study using a cohort of over 1,400 inpatients also revealed that diabetic patients with a history of metformin use have a significantly lower 3-year mortality than diabetic patients who have not taken metformin [12]. There are, however, conflicting reports as well. For example, the same effect was not observed in *Drosophila* [26]. Also, age-dependent, dose-dependent, and gender-dependent variable effects on lifespan were reported in mice [27, 28]. Although these previous studies’ results are not consistent, our cohort mentioned above (from which the present data are an analysis of its subgroup) clearly showed a positive influence of metformin use on survival among diabetic inpatients [12].

Our epigenetics data presented herein support metformin’s broad range of potential effects as indicated by the pathways identified through the enrichment analysis. The KEGG analysis (Table 7) showed several signals related to inflammation and the immune system, such as “interleukin-8 receptor activity” and “negative regulation of leukocyte apoptotic process.” The appearance of inflammation-related pathways is intriguing considering strong evidence showing that elderly people present with low-grade, chronic inflammation [29]. These signals identified in our study may support our hypothesis that metformin can modify the inflammatory process through epigenetic modification and influence the likelihood of survival. Consistent with our data, Barath et al. also reported that metformin inhibited cytokine production from Th17 by correcting age-related changes in autophagy and mitochondrial bioenergetics, indicating its potential for the medication to promote healthy aging [30]. Among the literature supporting metformin’s role in suppressing inflammation, clinical trials including the Diabetes Prevention Program (DPP) [31] and Bypass Angioplasty Revascularization Investigation 2 Diabetes (BARI 2D) [32] have provided further evidence of metformin’s role in changing inflammatory biomarker levels among diabetic patients, while other clinical trials, such as the Lantus for C-reactive Protein Reduction in Early Treatment of Type 2 Diabetes (LANCET) [33], have found opposing evidence. Although several studies mentioned here have investigated the relationship between metformin and its potential anti-inflammation, a clinical trial aimed to confirm metformin’s role in aging is yet to be seen [2, 34]. It is worth mentioning, nonetheless, a small clinical study that demonstrated the regression of epigenetic age of patients through the administration of recombinant human growth hormone (rhGH), dehydroepiandrosterone (DHEA), and metformin [15]. As the study team administered three medications to their subjects at the same time, it is impossible to distinguish epigenetic changes caused only by metformin. It is also worth mentioning the unexpected results from the Horvath epigenetic clock since subjects with history of metformin use had relatively higher age acceleration than subjects without history of metformin. Still, neither reached statistical significance (*p* < 0.05). Future prospective studies comparing epigenetics marks before and after metformin use would be needed to better understand the direct effect of the medication.

In DM-only subjects, A-kinase anchoring protein 13 (*AKAP13*) gene was found (Table 5). A recent study showed that AKAP13 inhibits mammalian target of rapamycin complex 1 (mTORC1), which was present in our enrichment analysis as “mTOR signaling pathway” (Table 3). Furthermore, the degree of *AKAP13* expression in lung adenocarcinoma cell lines correlates with mTORC1 activity [35]. Metformin’s anti-inflammatory effect has been shown to occur through eventual AMPK activation, which also inhibits the mTOR signaling pathway [18]. Metformin’s connection to AKAP13, which has yet been fully understood, deserves further investigation.

To the best of our knowledge, our study is the largest of its kind. A smaller, previous study also investigated metformin’s effect on genome-wide DNA methylation in human peripheral blood, although their study power was limited to a sample size of 32 male subjects [36]. Enrichment analysis in the present study revealing the longevity pathway from a hypothesis-free approach further strengthens our hypothesis that metformin exhibits its potential benefit for longevity through epigenetic processes. We also identified other relevant pathways associated with metformin’s mechanisms of action, such as the AMPK signaling pathway and HIF-1α signaling pathway [37].

Our study has several limitations. Although 171 subjects were analyzed retrospectively in this study, a controlled prospective study with a larger sample size would provide a better picture of the epigenetic mechanism of metformin on longevity. In addition, none of the individual CpG sites reached genome-wide significance (*p* < 5E-08). Thus, our findings should be interpreted as exploratory and hypothesis-generating. However, the fact that we found their biological relevance to metformin’s roles is still worth noting. As diabetes and metformin use status of the subjects was determined based on a retrospective chart review of electronic medical records, there are possibilities for misclassification, although we were still able to find multiple relevant pathways and genes of interest related to metformin’s action. Moreover, the duration of metformin use was not precisely assessed, making our definition of “metformin history use” broad since it might have included patients who took metformin for only a few months and patients who took metformin for years, for instance. Also, other types of diabetic medications were not investigated, such as sulfonylureas and glinide drugs as we used an already completed study dataset from our previous work. The rationale for us not investigating the influence of other diabetic medications was based on past literature showing that those diabetic medications other than metformin did not show benefits for survival. In fact, sometimes they were associated with worse mortality [38–40].

In summary, the data presented here support our hypothesis that epigenetics, especially DNA methylation, may be altered by metformin use and that such epigenetic processes potentially contribute to molecular mechanisms leading to longevity. Further careful investigation with a larger sample size would be warranted.

## METHODS

### Study participants and recruitment

We have previously recruited patients at the University of Iowa Hospital and Clinics (UIHC) for a separate study related to delirium from January 2016 to March 2020 [41–44]. Among them, we used data from a subgroup of patients recruited from November 2017 to March 2020 who had blood samples collected and processed for the epigenetics analysis [45–47]. Patients 18 years or older, who were admitted to the emergency department, orthopedics floor, general medicine floor, or intensive care unit were approached. Only those who consented, or whose legally authorized representative consented, were enlisted in the study. Written informed consent was obtained from all participants. Exclusion criteria included subjects whose goals of care were comfort measures only, those who were prisoners, or individuals with droplet/contact precautions. Further details of the study subjects and enrollment process are described previously [41–44].

We tested 173 subjects for genome-wide DNA methylation (DNAm) status, then conducted a post-hoc analysis of the available data to assess the influence of metformin. This study was approved by the University of Iowa Hospital and Clinics Institutional Review Board, and all procedures were compliant with the Declaration of Helsinki.

### Clinical information

Clinical variables were gathered through electronic medical chart review, patient interviews, and collateral information from family members [41–44]. Metformin use, insulin use, and type 2 diabetes mellitus (DM) history were obtained by using the search terms “metformin”, “insulin”, and “DM” or “diabetes”, respectively [12]. Only type 2 diabetes mellitus (DM) was included, excluding type 1 diabetes mellitus or gestational diabetes. If there was a history of metformin prescription before the study enrollment, patients were categorized as metformin users (Met). Those who were prescribed metformin after participation were not categorized as metformin users (non-Met) since the blood was obtained prior to such prescription.

### Sample collection

Blood samples were collected in EDTA tubes during patients’ hospital stay. Samples were shipped to the research laboratory and stored at −80°C until downstream analysis as a batch.

### Sample analysis

DNA was extracted from whole blood following the MasterPure™ DNA Purification kit (Epicentre, MCD 85201). DNA passing quality control based on NanoDrop spectrometry and in sufficient amount through the Qubit dsDNA Broad Range Assay Kit (ThermoFischer Scientific, Q32850) was selected for analysis for genome-wide DNAm status. 500 ng of genomic DNA from each sample was bisulfite-converted with the EZ DNA Methylation™ Kit (Zymo Research, D5002) and analyzed using Infinium HumanMethylationEPICBeadChip™ Kit (Illumina, WG-317-1002). The Illumina iScan platform scanned the arrays.

### Statistics and bioinformatics analysis

All analyses were conducted using R. The R packages ChAMP [48] and minfi [49] were used to process the data. Data from a total of 175 samples from 173 subjects were included for the statistical and bioinformatic analysis. DNAm levels for each CpG site were first compared between those with and without a history of metformin prescription (first run; Supplementary Table 1). Then, comparison limited among only DM patients between those with and without a history of metformin prescription was conducted to avoid potential influence of DM on DNAm status (second run; Supplementary Table 2).

During quality control processes, 2 samples from the first run and no samples from the second run were excluded based on the density analysis plots as a part of our quality control pipeline. 2 samples were also excluded because two patients had their blood collected twice. The first collected samples were included for further analysis while the second samples were excluded to maintain consistency between samples from all subjects. Therefore, 171 subjects from the first run and 63 subjects from the second run remained for the analysis. Furthermore, during the data loading process, probes were filtered out if they (i) had a detection *p*-value >0.01, (ii) had <3 beads in at least 5% of samples per probe, (iii) were non-CpG, SNP-related, or multi-hit probes, or (iv) were located on chromosome X or Y. Beta mixture quantile dilation [50] was used to normalize samples, while the combat normalization method was used to correct for batch effect in the first run [51, 52]. The second run, which only included diabetic patients, was not corrected for batch effect because there were individual patients who were not part of any batches.

Top hits based on each CpG site difference were obtained through the RnBeads package using the limma method [53, 54] and accounting for age, sex, insulin use, BMI and cell type proportions (CD8 T cells, CD4 T cells, natural killer cells, B cells, and monocytes) as covariates. DNAm Age Calculator available online [55] calculated the cell type proportions through the method reported previously [56].

After obtaining the top CpG sites, enrichment analysis followed using missMethyl [57] and unbalanced numbers of CpG sites on each gene were controlled using the EPIC array. Gene Ontology (GO) and Kyoto Encyclopedia of Genes and Genome (KEGG) [58] analysis was conducted. The number of CpG sites included in the analysis was determined by the combination of *p*-value and beta value cutoffs of the methylation rates of each CpG site (*p* < .01 and beta >0.04). Genome-wide significance was set at a *p*-value of less than = 5.0E-08.

The chi-square test compared the categorical data (sex, race, and insulin use) between two groups, while the Welch’s *t*-test compared the numerical data (age, BMI, and CCI) between two groups.

### DNA methylation aging clock analysis

To investigate whether subjects with history of metformin use had slower “age acceleration” than subjects without history of metformin use, we submitted the raw DNA methylation beta values to a publicly available tool, which includes the Horvath [55] method. The calculated output was the difference between the DNA methylation age and the chronological age.

### Availability of data materials

The datasets analyzed during the current study are available from the corresponding author upon reasonable request.

## Supplementary Materials

Supplementary Table 1

Supplementary Table 2
